# Supplementary material for: Proteomic patterns associated with response to breast cancer neoadjuvant treatment
Source: Mol Syst Biol. 2020 Sep 22;16(9):e9443. doi: 10.15252/msb.20209443 (PMC7507992; doi:10.15252/msb.20209443)
Supplement: Supplementary file 1 — Appendix [file MSB-16-e9443-s001.pdf]

Table of contents

Appendix Figure S1: Unsupervised analysis of proteomics data.

Appendix Figure S2. WGCNA dendrogram and eigengene modules.

Appendix Figure S3. Protein network of WGCNA modules associated with Tumor size and relapse.

Appendix Figure S4. PYCR1 and Survival.

Appendix Figure S5. CRISPR Cas9 based knockout of PYCR1 and effect on Proline levels.

Appendix Figure S6. CRISPR Cas9 based knockout of PYCR1 in MCF7 cells in-vivo.

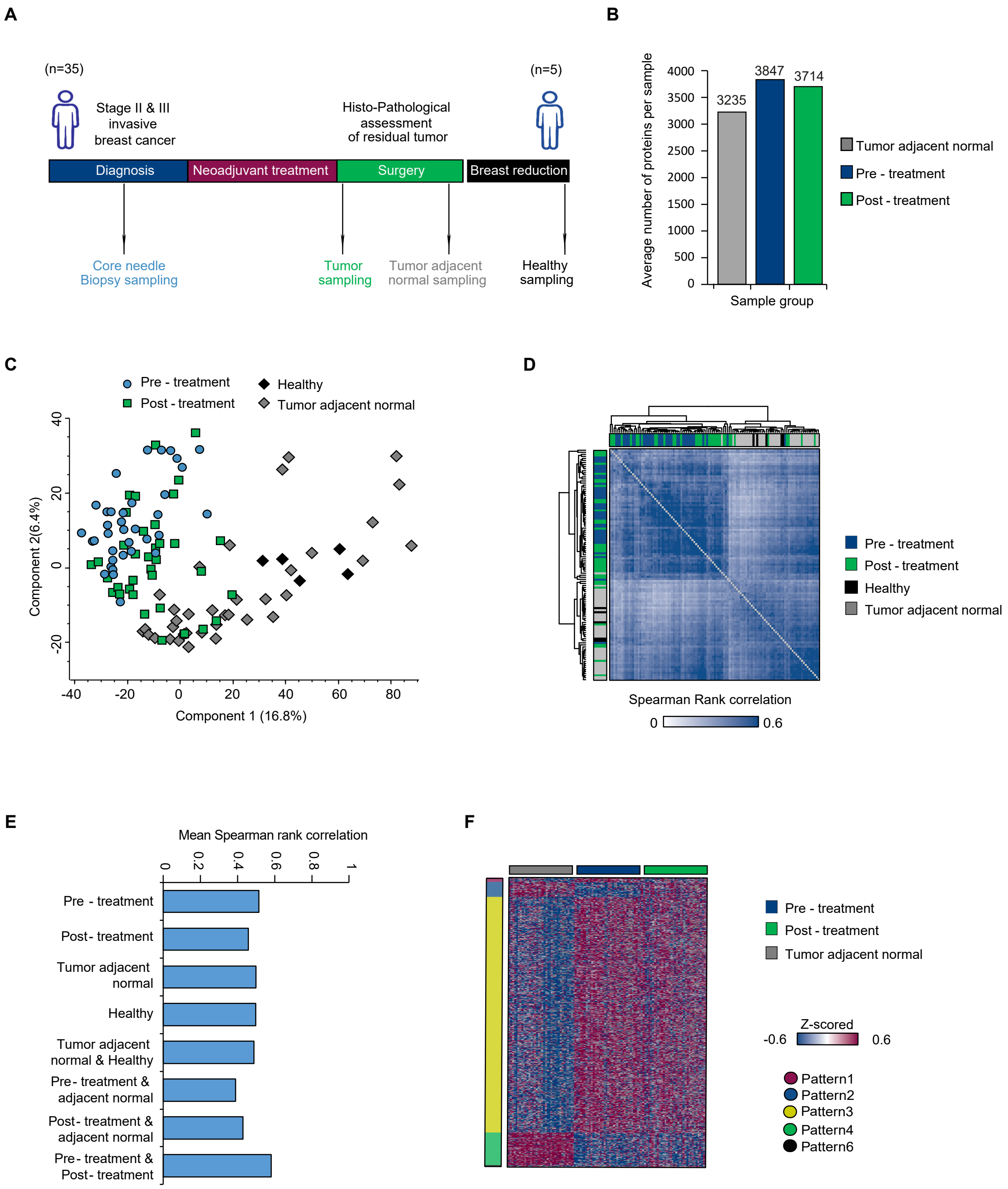

Appendix Figure S1: Unsupervised analysis of proteomics data. Related to Figure1.

A) Neoadjuvant cohort assembly and sampling workflow for proteomic analysis. Three matched samples were obtained from each patient, including tumor adjacent normal tissue, pre-treatment and post-treatment tumor samples. **B)** Average number of proteins quantified in each group. **C)** Principal component analysis of all samples in the cohort (n=113) including five healthy samples from breast reduction surgery shows good separation between tumor and normal samples. **D)** Mean Spearman rank correlations of all 113 samples. Color bars indicate sample type. **E)** Bar plot indicates average correlation of all samples within each group and average correlation of matched samples between groups. **F)** Heatmap of 904 significantly changing proteins that follow five patterns of protein abundance dynamics. Patterns were selected using paired Student's t-test with FDR 5% between matched samples (global pattern analysis).

A

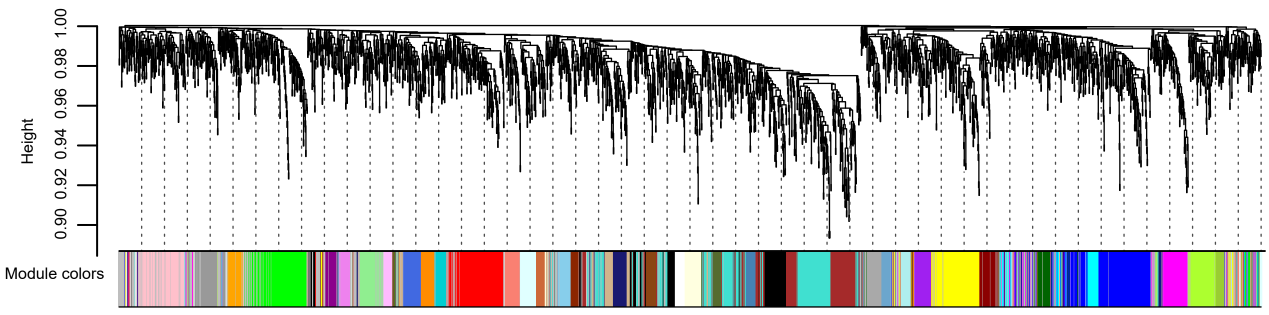

B

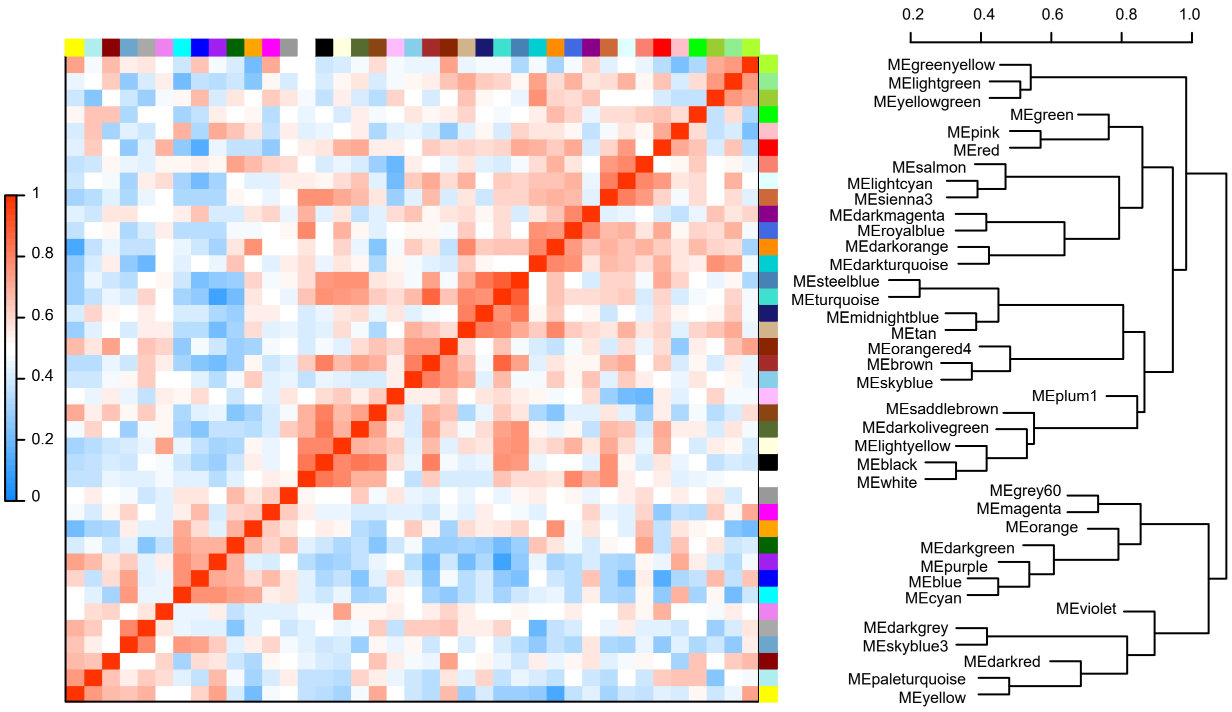

Appendix Figure S2. WGCNA dendrogram and eigengene modules. Related to Figure 3.

A) Dendrogram showing 39 protein modules determined by WGCNA. Modules are indicated by module colors. **B)** Heatmap of eigengene similarity matrix and associated dendrogram. Modules are indicated by module colors.

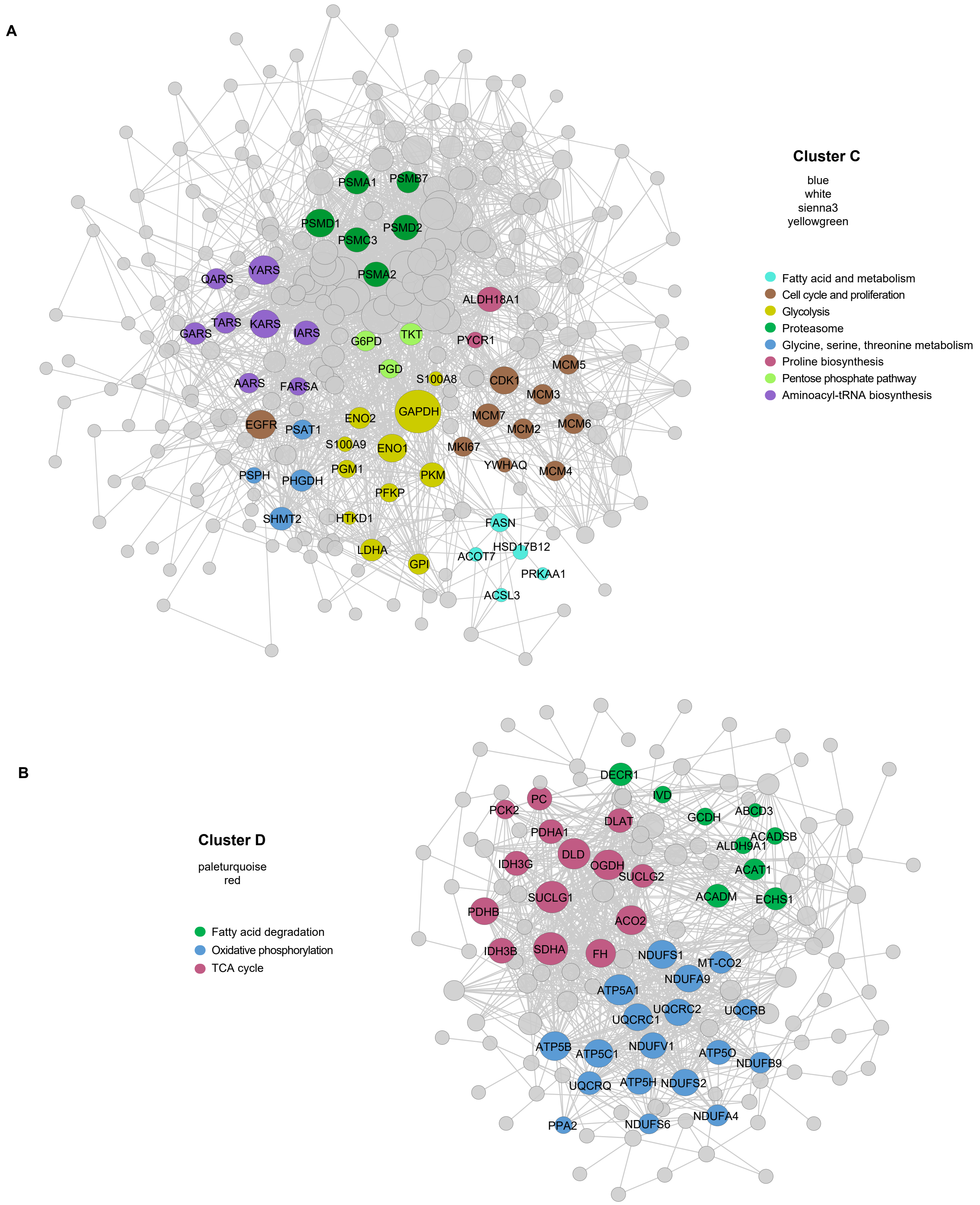

Appendix Figure S3. Protein network of WGCNA modules associated with Tumor size and relapse. Related to Figure 3 and 4.

A) Protein networks of all cluster C proteins are shown. Networks were constructed using the STRING database and connected nodes were visualized in Cytoscape. Node size is based on degree of connectivity of each protein to other interacting proteins (minimum 1, maximum 88). Node colors represent different biological pathways as indicated. **B)** Same as (**A**) for cluster D.

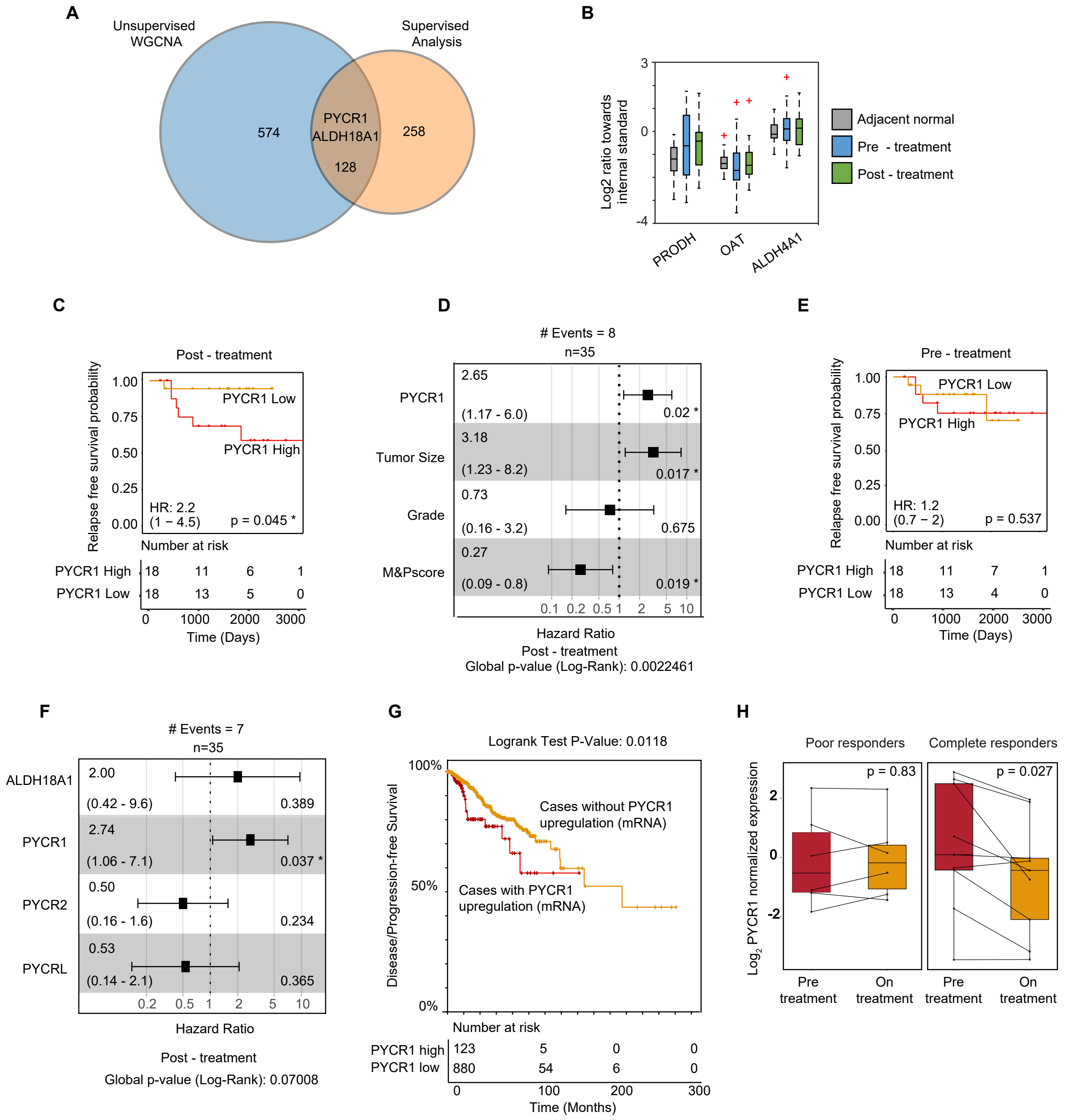

Appendix Figure S4. PYCR1 and Survival. Related to Figure 4.

A) Venn diagram showing overlap of proteins associated with either relapse or M&P score between supervised and unsupervised WGCNA analysis. PYCR1 and ALDH18A1 are among the 128 proteins that are shared between the two analyses. **B)** Abundance levels of proline metabolism proteins. OAT, Ornithine amino transferase; ALDH4A1, Aldehyde Dehydrogenase 4 Family Member A1; PRODH, Proline oxidase. **C)** Kaplan Meier survival curve for post-treatment PYCR1 levels in residual cancer. Cox univariate p value and hazard ratio with 95% CI indicated. Protein level was defined as low /high based on the median abundance value. **D)** Cox multivariate analysis for post-treatment levels of PYCR1 and confounding factors such as tumor grade, tumor size and M&P score. p value and hazard ratio with 95% CI is indicated. Number of events is 8 and all 35 patients are included in the analysis. **E)** Same as **(C)** but for pre-treatment PYCR1 levels. **F)** Cox multivariate analysis for post-treatment levels of proline biosynthesis genes. p value and hazard ratio with 95% CI is indicated. Number of events is 7 and all 35 patients are included in the analysis. **G)** Kaplan Meier survival curve for Disease free progression when PYCR1 is either upregulated (>2 SD from mean of expression in the reference population) or not upregulated (<2 SD from mean of expression in the reference population) on the mRNA level as measured by RNA Seq V2 RSEM. Data comprises of 1010 samples from the TCGA invasive breast cancer provisional dataset. Data was visualized using the cBioportal platform. **H)** Log2 Normalized PYCR1 level (TMT reporter ion intensity) differences between matched Pre and On treatment samples (72 hrs after initiation of neoadjuvant chemotherapy). Patients are separated based on the overall response to Neoadjuvant chemotherapy. Paired samples are compared using Wilcoxon-rank sum test.

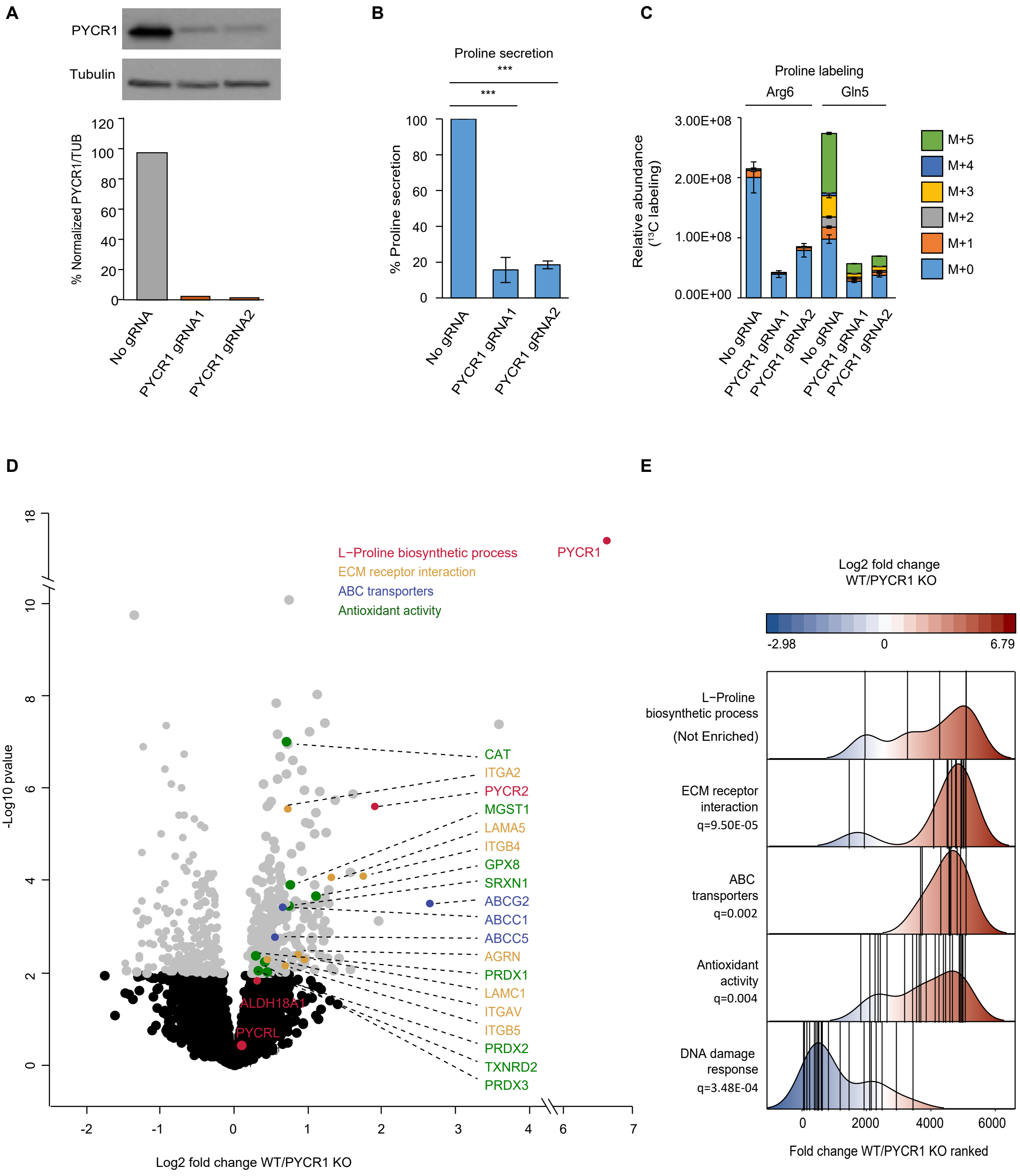

Appendix Figure S5. CRISPR Cas9 based knockout of PYCR1 and effect on Proline levels. Related to Figure 5 and 6.

A) Western blot showing PYCR1 knockout with two guide RNAs. Bar plot shows quantitative analysis of the western blot. **B)** PYCR1 knockout was validated by analyzing extracellular proline secretion into the cell culture medium. Data represent normalized mean ± SE of three biological experiments compared using Kruskall Wallis test followed by Dunett's test for multiple pairwise comparisons. Corrected p values are indicated as follows $^*p < 0.05$, $^{**}\ p < 0.01$, and. $^{***}\ p < 0.001$. **C)** PYCR1 knockout was validated by analyzing $^{13}C_5$ Glutamine and $^{13}C_6$ Arginine derived carbon labeling of proline. Data are presented as mean ± SD of triplicate samples. **D)** Volcano plot highlights the significantly changing proteins (grey, BH FDR 10%) between WT and PYCR1 KO MCF7 cells. Proteins that are significantly downregulated in the PYCR1 knockout cells are highlighted. Colors indicate different biological processes. **E)** Ridge plot shows distribution of ranked proteins (Lowest fold change WT/KO to highest). Processes were selected based on 1D annotation enrichment performed on the ratios of WT vs. KO cells. BH FDR q-values are indicated.

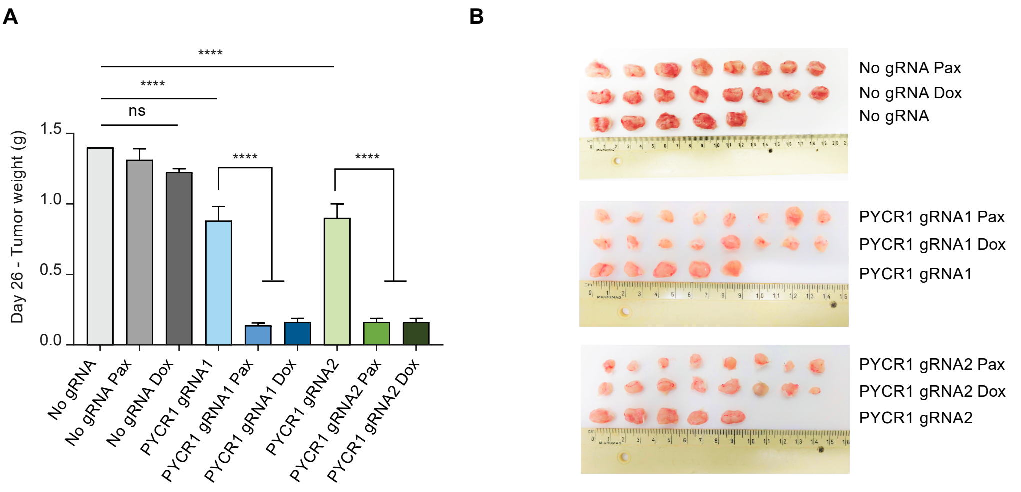

Appendix Figure S6. CRISPR Cas9 based knockout of PYCR1 in MCF7 cells in-vivo. Related to Figure 6.

A) Bar plot indicates mean ± SE of tumor weight measurements for day 26. Groups are compared by one way ANOVA followed by Tukey's multiple comparisons test for pairwise group comparisons. Corrected p-values are reported as follows *p < 0.05, ** p < 0.01, *** p < 0.001,**** p<0.0001. **B)** Picture of excised tumors on day 26.
