# Supplementary material for: Proteomic patterns associated with response to breast cancer neoadjuvant treatment
Source: Mol Syst Biol. 2020 Sep 22;16(9):e9443. doi: 10.15252/msb.20209443 (PMC7507992; doi:10.15252/msb.20209443)
Supplement: Supplementary file 2 — Expanded View Figures PDF [file MSB-16-e9443-s002.pdf]

Expanded View Figures

Figure EV1. Supervised analysis. Related to Fig 3.

A Networks of all proteins that are significantly upregulated in patients with good prognosis and unaltered in patients with poor prognosis. Networks were constructed using the STRING database, and connected nodes were visualized in Cytoscape. Node color is based on the association to clinical feature. Selected significantly enriched GO and KEGG biological pathways are indicated by node border colors (Fisher exact test, FDR 5%).

B Same as (A) but for all proteins that are significantly downregulated in patients with good prognosis and unaltered in patients with poor prognosis.

© 2020 The Authors

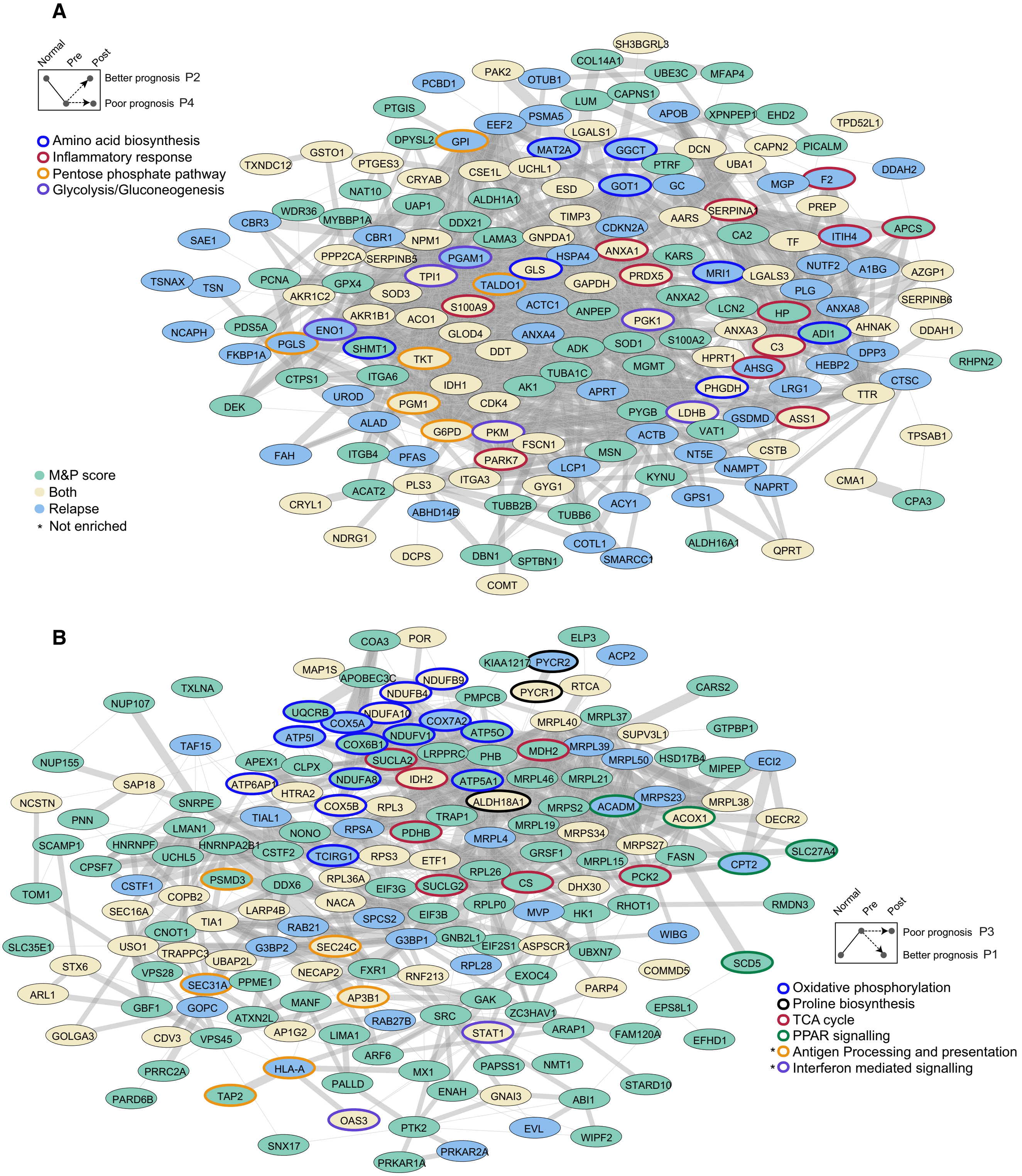
A
Normal
Pre
Post
Better prognosis P2
Poor prognosis P4
Amino acid biosynthesis
Inflammatory response
Pentose phosphate pathway
Glycolysis/Gluconeogenesis
M&P score
Both
Relapse
* Not enriched
SH3BGRL3
COL14A1
PAK2
OTUB1
UBE3C
MFAP4
PCBD1
CAPNS1
LUM
PTGIS
PSMA5
APOB
XPNPEP1
EHD2
TPD52L1
EEF2
LGALS1
DPYSL2
GPI
MAT2A
GGCT
DCN
CAPN2
PICALM
GSTO1
PTGES3
UCHL1
PTRF
UBA1
TXNDC12
CRYAB
CSE1L
GOT1
GC
MGP
F2
DDAH2
NAT10
ESD
UAP1
ALDH1A1
WDR36
PREP
SERPINA1
CBR3
MYBBP1A
DDX21
TIMP3
AARS
SAE1
CBR1
NPM1
LAMA3
GNPDA1
CDKN2A
CA2
TF
ITIH4
APCS
PPP2CA
SERPINB5
PGAM1
HSPA4
ANXA1
KARS
NUTF2
A1BG
AZGP1
TSNAX
GLS
TPI1
GAPDH
PRDX5
MRI1
LGALS3
TSN
PCNA
GPX4
TALDO1
PLG
SERPINB6
AKR1C2
SOD3
ANXA2
S100A9
ACTC1
ANPEP
LCN2
ANXA8
HP
AKR1B1
ACO1
PGK1
AHNAK
DDAH1
NCAPH
PDS5A
ENO1
GLOD4
ANXA4
ANXA3
ADI1
PGLS
S100A2
SOD1
C3
FKBP1A
SHMT1
ADK
DPP3
DDT
TKT
HPRT1
RHPN2
MGMT
AHSG
HEBP2
CTPS1
ITGA6
TUBA1C
IDH1
AK1
PHGDH
LRG1
CTSC
APRT
UROD
TTR
DEK
PGM1
CDK4
PYGB
LDHB
GSDMD
ASS1
ALAD
VAT1
ACTB
G6PD
PKM
TPSAB1
FSCN1
FAH
ITGB4
MSN
NT5E
CSTB
PFAS
PARK7
LCP1
NAMPT
KYNU
CMA1
ITGA3
PLS3
ACAT2
GYG1
NAPRT
CPA3
CRYL1
ACY1
GPS1
TUBB2B
ABHD14B
TUBB6
NDRG1
COTL1
ALDH16A1
QPRT
DCPS
DBN1
SPTBN1
SMARCC1
COMT
B
COA3
POR
ELP3
KIAA1217
PYCR2
ACP2
MAP1S
APOBEC3C
NDUFB9
RTCA
CARS2
TXLNA
NDUFB4
PYCR1
NUP107
PMPCB
UQCRB
NDUFA10
MRPL37
MRPL40
COX5A
COX7A2
SUPV3L1
GTPBP1
ATP5I
NDUFV1
ATP5O
COX6B1
TAF15
LRPPRC
PHB
MDH2
MRPL39
NUP155
APEX1
SUCLA2
HSD17B4
MRPL50
ECI2
CLPX
MIPEP
SAP18
ATP6AP1
NDUFA8
IDH2
ATP5A1
MRPL46
MRPL21
HTRA2
MRPS23
NCSTN
SNRPE
COX5B
RPL3
ALDH18A1
MRPL38
TRAP1
MRPS2
ACADM
PNN
TIAL1
ACOX1
DECR2
LMAN1
RPSA
NONO
PDHB
MRPL19
MRPS34
MRPL4
SCAMP1
HNRNPF
HNRNPA2B1
CSTF2
MRPS27
SLC27A4
GRSF1
UCHL5
TCIRG1
RPS3
FASN
CPT2
CPSF7
ETF1
MRPL15
RPL26
PSMD3
RPL36A
PCK2
CSTF1
DDX6
CS
DHX30
TOM1
EIF3G
SUCLG2
COPB2
RHOT1
RMDN3
NACA
RPLP0
MVP
SEC16A
LARP4B
HK1
TIA1
SPCS2
EIF3B
RAB21
WIBG
CNOT1
GNB2L1
SLC35E1
USO1
G3BP1
EIF2S1
ASPSCR1
TRAPPC3
G3BP2
SEC24C
UBXN7
SCD5
UBAP2L
RPL28
STX6
VPS28
NECAP2
FXR1
COMMD5
EXOC4
RNF213
SEC31A
PPME1
PARP4
ARL1
AP3B1
GAK
MANF
GBF1
GOPC
EPS8L1
ZC3HAV1
SRC
ATXN2L
RAB27B
ARAP1
AP1G2
FAM120A
GOLGA3
CDV3
VPS45
LIMA1
STAT1
EFHD1
ARF6
PRRC2A
HLA-A
MX1
PAPSS1
NMT1
PALLD
STARD10
ENAH
GNAI3
PARD6B
TAP2
ABI1
OAS3
PTK2
EVL
WIPF2
SNX17
PRKAR2A
PRKAR1A
Normal
Pre
Post
Poor prognosis P3
Better prognosis P1
Oxidative phosphorylation
Proline biosynthesis
TCA cycle
PPAR signalling
* Antigen Processing and presentation
* Interferon mediated signalling

Figure EV1.

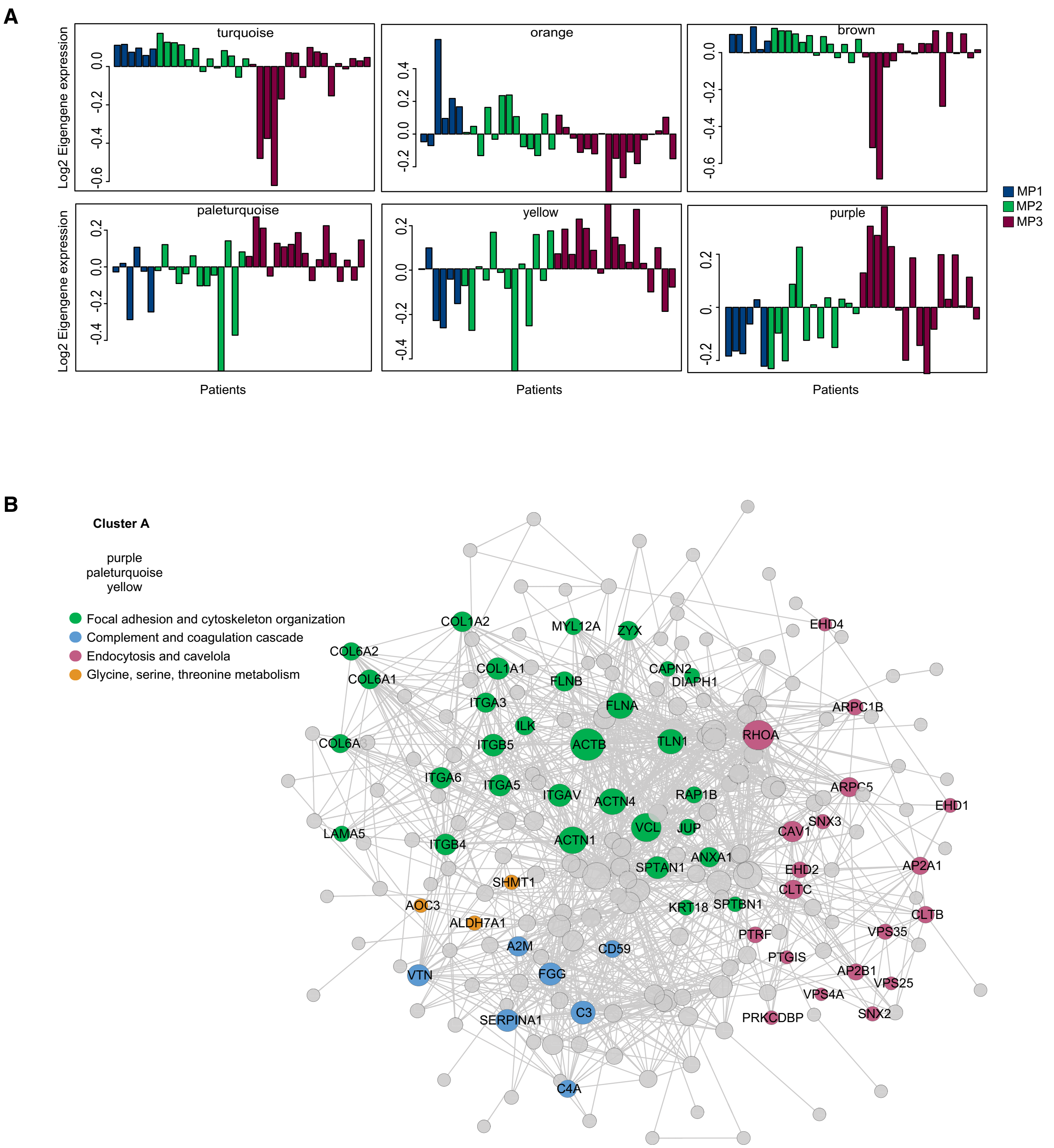

Figure EV2. Module eigengene profiles associated with M&P score. Related to Fig 3.

A Module eigengene profiles of protein modules in post-treatment samples that show a significant correlation to M&P score. Each bar represents a patient and is sorted and colored according to M&P score.

B Protein networks of all cluster A proteins are shown. Networks were constructed using the STRING database, and connected nodes were visualized in Cytoscape. Node size is based on degree of connectivity of each protein to other interacting proteins (minimum 1, maximum 88). Node colors represent different biological pathways as indicated.

 © 2020 The Authors

Figure EV3. Protein network of WGCNA modules associated with M&P score. Related to Fig 3.

A Protein networks of all cluster B proteins. Networks were constructed using the STRING database, and connected nodes were visualized in Cytoscape. Node size reflects degree of connectivity of each protein to other interacting proteins. Node colors represent different biological pathways as indicated.

B Global pattern proteins in cluster A (combined protein list of WGCNA modules, turquoise, orange, and brown), or in cluster B (combined protein list of pale-turquoise, yellow, and purple modules). Patients are separated by M&P response score. Heatmap shows average protein levels in each sample type. Global pattern of protein levels and WGCNA module in each response group is indicated as row annotation.

▶

© 2020 The Authors

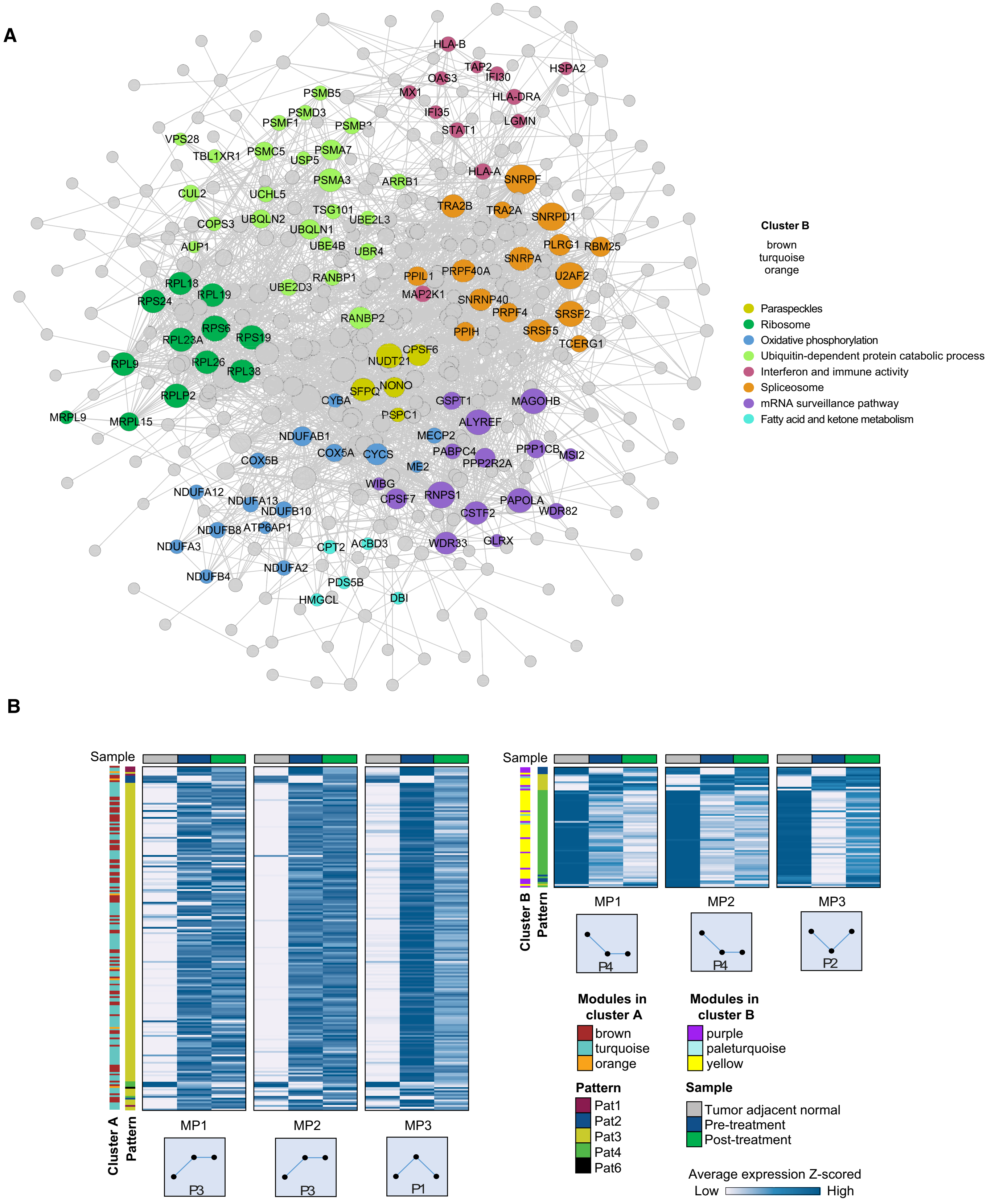

Figure EV3.

 © 2020 The Authors

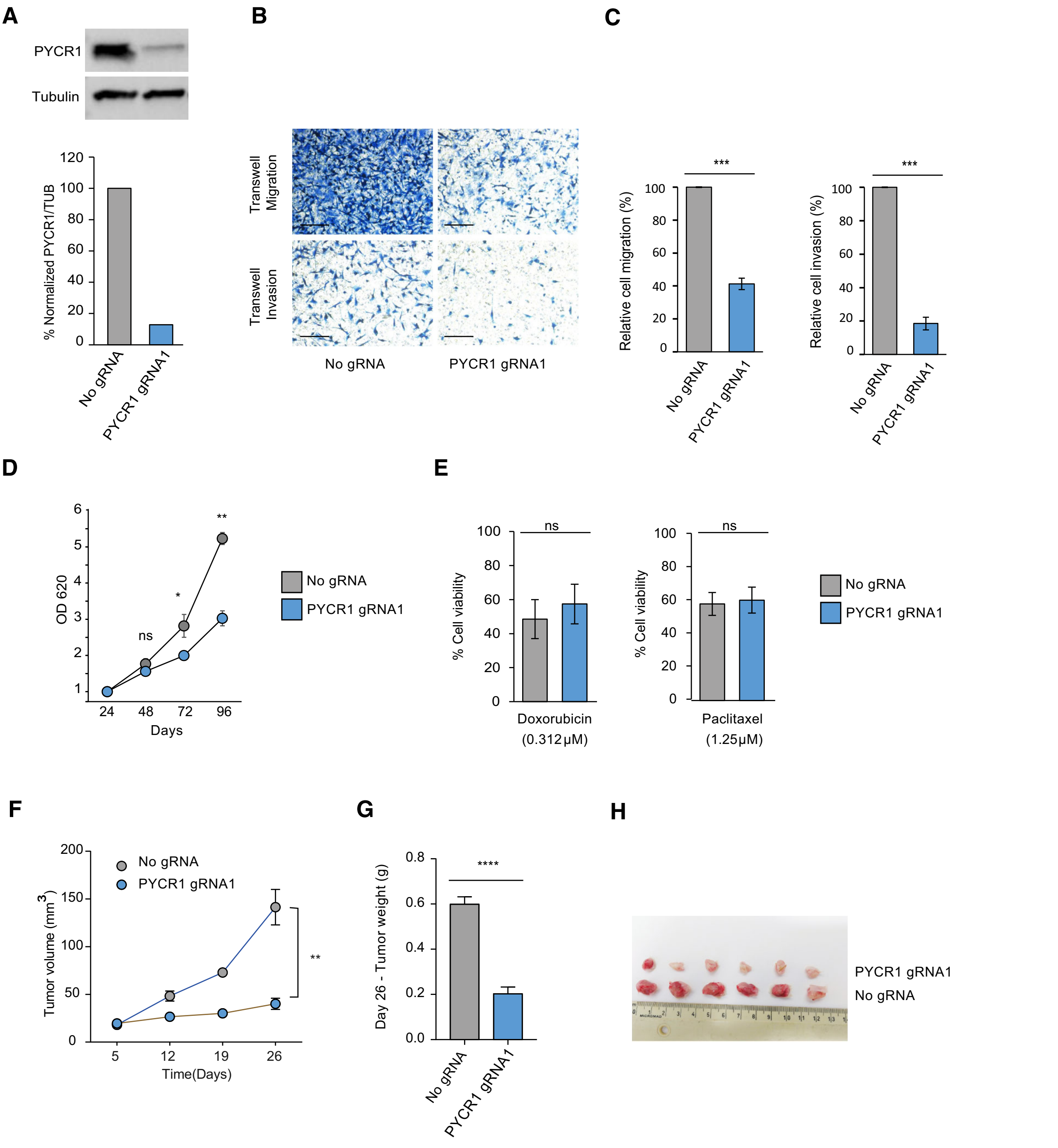

Figure EV4. CRISPR Cas9-based knockout of PYCR1 in MDA-MB-231 cells. Related to Fig 6.

- A Western blots showing PYCR1 knockout in MDA-MB-231 cells. Bar plot shows quantitative analysis of the Western blot.
- B Representative pictures of Transwell migration and invasion after PYCR1 knockout. Scale bar: 100 µm.
- C Bar plots represent mean ± SD of three biological replicates of the invasion and migration assays. Samples are compared using paired Student's *t*-test. *P* values are indicated as ***$P < 0.001$.
- D Growth measurements of MDA-MB-231 wild-type and PYCR1 KO cells over 96 h. Data represent mean ± SE of three biological replicates. Samples are compared using paired Student's *t*-test. *P* values are indicated as follows *$P < 0.05$ and **$P < 0.01$.
- E Bar plots show percentage of viable cells after treatment with 0.312 µM doxorubicin and 1.25 µM paclitaxel. Data represent mean ± SE of three biological experiments. Samples are compared using Student's *t*-test.
- F Tumor volume measurements for 26 days in MDA-MB-231-injected NSG mice. CRISPR control ($n = 6$) and PYCR1 KO tumors ($n = 6$) without treatment are shown. Data represent mean ± SE. Samples on day 26 are compared using paired Student's *t*-test, and *P* values are reported as follows **$P < 0.01$.
- G Bar plot indicates mean ± SE of tumor weight measurements for day 26. CRISPR control ($n = 6$) and PYCR1 KO tumors ($n = 6$) without treatment are shown. Samples are compared using paired Student's *t*-test. *P* values are reported as **** $P < 0.0001$.
- H Picture of excised tumors on day 26.
